# Supplementary material for: Comparison of Motor Difficulties Measured in the First Year of School among Children Who Attended Rural Outdoor or Urban Conventional Kindergartens
Source: Int J Environ Res Public Health. 2022 Oct 29;19(21):14158. doi: 10.3390/ijerph192114158 (PMC9653742; doi:10.3390/ijerph192114158)
Supplement: Supplementary file 1 [file ijerph-19-14158-s001.zip › ijerph-1971248-supplementary.pdf]

STASTISTICAL ANALYSIS PLAN - 09-06-2022

Study title

Differences in attained motor skills tested after school entry among children who attended outdoor or conventional kindergartens

Suggested authors

Ina Olmer Specht, Sofus C Larsen, Jeanett Friis Rohde, Jane Nautrup Østergaard, Berit Lilienthal Heitmann

Purpose

In this statistical analysis plan, we describe the background and rationale for our study, in addition to general aspects of data preparation, sample size considerations and data analysis. The plan was set up and circulated with all authors prior to conducting any statistical analyses.

1 Background

A sedentary lifestyle with long time spent indoor and/or in front of a screen is becoming more and more the norm even in children as young as 3-5 years, especially among those from urban areas or less affluent families (1). Studies have shown that this unhealthy lifestyle of pre-schoolers, co-occurring with a low level of physical activity, seems related to both physical health problems and to low self-esteem (2). Most pre-school children in Denmark attend up to 40 hours of kindergarten per week (3). This makes kindergartens to institutions that contribute substantially to the daily level of physical activity and thus an ideal setting for finding ways to improve active outdoor lifestyles as a public health initiative.

In the Scandinavian countries there are overall two types of kindergartens with the same rating of personnel per child; 1) the conventional kindergarten where children spend time both indoor playing with toys, drawing and tumbling, and outdoor in the kindergarten playground, and 2) the outdoor kindergartens, a Danish invention from 1952, where almost all hours during the day are spend outdoor, usually in forests or in rural areas without a formal playground (4-6). Children attending outdoor kindergartens are often living in urban areas and are thus transported by bus from the city to the rural kindergarten setting.

Positive opinions in relation to health, physical activity and motor development are commonly reported by the media for outdoor kindergarten children (7). The evidence for these health benefits is, however, limited and only a few small Scandinavian studies have attempted to investigate this. For instance, a Danish study investigated motor development, level of awareness, ingenuity and frequency of sickness absence among children attending one outdoor kindergarten group compared to one conventional kindergarten group (8). In this study, the children from the two types of kindergartens were examined over a 10 months period. Results showed that children from the outdoor kindergarten scored better on all outcomes examined. Similarly, a study among 5-7 years old kindergarten children in Norway investigated

versatile play in an outdoor forest environment (n=46 children) compared to a kindergarten playground (n=29 children) (9). This study showed that over a period of 9 months, the children who daily played 1-2 hours in the forest gradually improved their motor ability more than the children who spent 1-2 hours daily at the kindergarten playground (9). A systematic review including 296 studies evaluated the evidence for relationships between nature contact and children's health (10). The majority of the included studies were cross-sectional, investigating residential green spaces by land-use data or Normalized Difference Vegetation Index, in relation to physical activity, of which 66% of the studies reported positive associations between nature and physical activity (10). The uneven ground, and more room for ingenuity have been proposed as the main factors explaining the higher physical activity level and motor skills observed in children playing in outdoor environments (9, 11).

Common to many studies that have examined health benefits of outdoor kindergartens and nature are that in addition to having examined small samples of children only, many of them had a high risk of selection or confounding bias due to lack of comparability among investigated groups (10). In this regard, we previously showed in a large register-based study that children who attended outdoor kindergartens differed according to parental socio- and early childhood demographics as compared to children attending conventional kindergartens (Specht et al. in prep.). Outdoor kindergarten children come from higher socio demographic families which can cause selection bias and are thus highly relevant to consider when investigating health outcomes related to kindergarten type attainment (Specht et al. in prep.).

In Denmark, all children attend a health nurse examination during the first year of school, often including a motor ability test. These measures combined with the register-based information on parental socio- and early childhood demographics, gives us a unique opportunity to examine outdoor kindergartens as a potential early preventive strategy towards better motor skills.

Our primary objective is thus to examine whether children in outdoor kindergartens have a lower risk of motor difficulties measure after school entrance compared to children from conventional kindergartens. Motor skills were assessed based on the number of remarks in the first available mandatory motor test made by the school health nurse often during the first year of school.

We hypothesise that compared to children attending conventional kindergartens, children attending outdoor kindergartens have long-term health benefits that persists after leaving kindergarten and include better motor skills, due to more physical activity in the outdoor kindergarten.

2 Material and Methods

The present study is part of the 'Outdoor kindergartens - the healthier choice?' (ODIN) study which was initiated with the main goal to investigate pedagogic and didactic practise as well as health effects including physical activity, sleep and infections requiring antibiotics and long-term outcomes e.g. weight, growth and motor development among children attending outdoor kindergartens compared to children

attending conventional kindergartens. In the ODIN study we have data on a total of 5526 children, of which 2651 attended outdoor kindergartens and 2875 attended traditional kindergartens, based on data from the Copenhagen Municipality and Aarhus Municipality, the two largest cities of Denmark. Both municipalities provided personal identification numbers (CPR-numbers) and health information gathered by health nurses during the first year of the child's life and from the school health surveys from all children who went to an outdoor kindergarten in the period 2011-2019, and from a random subsample of all children from conventional kindergartens, from the same areas of parental residence. This data was merged with data from the Danish registers to gather baseline demographic and health information on parents and children. In the present study motor development was only available from Copenhagen Municipality and in the years 2014-2019 since Aarhus Municipality do not mandatory test for motor development. We will thus include all children who have information on motor skills recorded after school entry (n=2093) without missing co-variates (n missing= 199) n=1894 children.

2.1.1 Exposure assessment

The exposure is attendance of an outdoor kindergarten or a conventional kindergarten. The participants had to have attended either an outdoor kindergarten or a conventional kindergarten in the years they lived in the municipality, thus the exposure time might differ according to how many years the child lived in the municipality. Type of kindergarten and when the child was admitted or ended kindergarten were registered by the municipalities, saved in a database and available to this study.

2.1.2 Outcome assessment and covariates

The outcome is the first mandatory motor test recorded after school entry, approximately at age 6 years. The motor test includes six sub-tests; 'dynamic jump on the spot', 'handle a writing tool', 'cutting with a scissor following a line', 'standing on one leg for 10 seconds on each leg', 'throwing' and 'grabbing'. Each test can get one remark if not managed by the child. In the present study, motor difficulties are defined based on having two or more remarks from the total of the six sub-tests. This number of remarks was chosen based on results from a Danish report investigating motor development among 5963 children from 13 Danish municipalities (not including Copenhagen and Aarhus) after school entry in the school year 2018/19 (12). In the report nine motor tests were performed and having three or more remarks were considered as motor difficulties. The municipalities included in the report have nine motor tests whereas the Copenhagen Municipality only have six tests. Since one remark also can have consequences on the child's everyday life (12), we also investigate this.

To gather information on covariates, CPR and health nurse data including motor skills, weight and height were sent to Statistics Denmark where merging at the individual level with information from The Danish Medical Birth Register and population data from Statistic Denmark took place.

Potential confounders/covariates:

- Preterm birth (born before GW 37, yes/no)
- Maternal education (Basic [basic school 8th–10th class]; Short [general upper–secondary education, short-cycle higher education or vocational education and training]; Medium [medium-cycle higher education or bachelor]; and Long [long-cycle education and PhD])
- Sex (female/male)
- Maternal origin (Western/non-Western)
- Birth wight (continuous, g)
- Age at motor test (continuous, years)

2.1.3 Statistical power and precession

Assuming 947 children in each group and 8.4% children with motor difficulties in the control group, we will have more than 80% power to detect odds ratios < 0.6 or > 1.5 (Figure S1). With 96% certainty the estimated width of 95% CIs for ORs above 1.5 will be below 1.9.

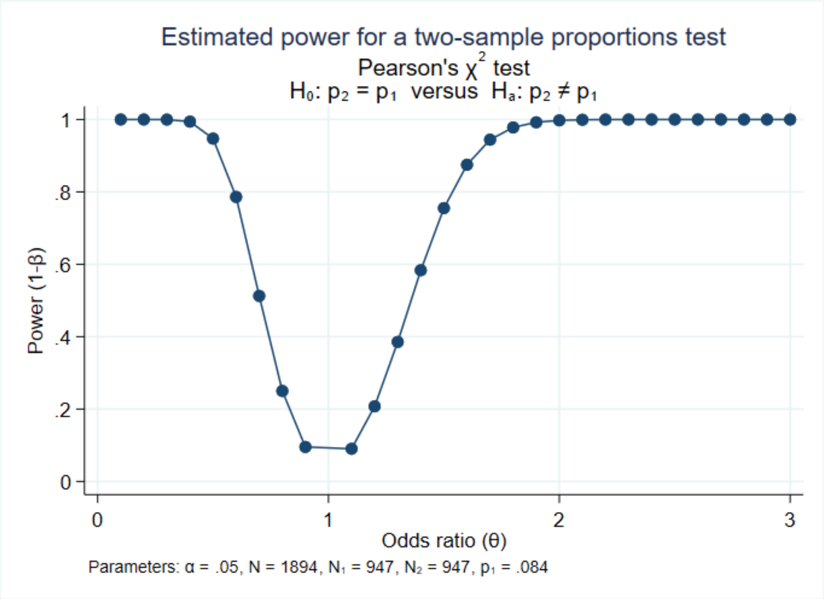

Figure S1. Estimated power for the primary analysis.

2.1.4 Statistical analysis

Primary analysis

Logistic regression will be used to examine the difference in motor development defined as two or more remarks (yes/no) between the two groups based on the first recorded test after school entry. Results

will be presented from crude analyses with information on motor development and kindergarten type only, and fully adjusted with added information on all additional covariates described above.

Sensitivity analysis

Similarly to the primary analysis, we will investigate motor difficulties related to having one or more remarks (yes/no), only, both in crude and fully adjusted analyses.

Sex is a predictor of motor development, further, analyses from the ODIN project have shown sex differences in choosing type of kindergarten, with more boys attending outdoor kindergartens. Effect modification by sex will be investigated by adding the sex variable and a product term (*kindergarten type × Sex*) to the fully adjusted model after removing the original sex variable from the model. If interactions are found, we will conduct subgroup analyses specifically by sex.
Also, SES is a predictor of childhood physical activity and potentially related to motor development, further, analyses from the ODIN project have shown parental education to be higher in outdoor kindergartens. Thus, in addition to adjustment for maternal education, we will investigate effect modification by SES by adding the SES variable and a product term (*kindergarten type × SES*) to the fully adjusted model after removing the original maternal education variable from the model. If interactions are found, we will conduct subgroup analyses specifically for children of low SES mothers (defined as mothers with a basic or short education) and high SES mothers (defined as mothers with a medium or long education).

Finally, we will examine a potential dose–response relationship using information on the total time in years spend in kindergarten (continuous variable) in relation to motor difficulties and test the effect modification by type of kindergarten by adding a product term to the model (years in kindergarten x type of kindergarten).

All statistical tests will be two-sided with a significance level at 0.05. All statistical analyses will be performed using SAS or STATA.

2.2 Ethical considerations

Permission from the municipalities to send information to Statistics Denmark was granted. Permission from the Ethical Committee was evaluated not to be relevant (journal nr.: H-19053587). Permission from the Capital Region Data Agency and the Danish Patient Safety Authority was granted (Journal nr.: P-2020-54 and 31-1521-8, respectively). Registries relevant for the study are accessible and the collection of data from these registries is in accordance with accepted ethical principles for informed consent according to the Declaration of Helsinki.

3 Results

Templates of figures and tables are presented in this section. Some additional sensitivity analyses may be added subsequently.

Table S1. Participant characteristics of children in outdoor kindergartens versus conventional kindergartens. Results are presented as percentage or mean (SD).

	Outdoor kindergarten n=			Traditional kindergarten n=		
	n	%	Mean (SD)	n	%	Mean (SD)
Female sex						
Age at kindergarten enrolment, years						
Age at first school motor measurement, years						
Time spent in kindergarten, days						
School entry motor test, tests with at least one remark						
School entry motor test, tests with at least two remarks						
Birth weight, g						
Preterm birth						
Maternal education						
Basic						
Short						
Medium						
Long						
Paternal education						
Basic						
Short						
Medium						
Long						
Maternal country of origin						
Western						
Non-Western						
Paternal country of origin						
Western						
Non-Western						

Table S2. Motor skills after school entry among children from outdoor versus conventional kindergartens.

	OR	95% CI
Motor, ≥2 remarks		
Crude		
Adj. model 1[1]		
Motor, ≥1 remark		
Crude		
Adj. model 1[1]		

[1] Adjusted for sex, birth weight, preterm birth, maternal education, maternal origin, age at motor test.

4 References

1. Gable S, Chang Y, Krull JL. Television watching and frequency of family meals are predictive of overweight onset and persistence in a national sample of school-aged children. J Am Diet Assoc. 2007;107(1):53-61.
2. Timmons BW, Naylor PJ, Pfeiffer KA. Physical activity for preschool children--how much and how? Can J Public Health. 2007;98 Suppl 2:S122-34.
3. Jessen FB. Flere børn er i institution længere tid end deres forældre er på job. Kristeligt Dagblad. 2015 19.09.2015.
4. Söderström MM, F; Grahn, P; Blennow, M;. Utomhusmiljön i Förskolan. Betydelse för lek och utevistelse. Ugeskr Laeger. 2004;166/36.
5. Christoffersen MH-S, AK; Laugesen, L;. Daginstittionens Betydning for Børns Udvikling, en forskningsoversigt. Copenhagen: SFI Det nationale Forskningscenter for velfærd; 2014.
6. Forening DP-H. Udflytterbørnehaver. Små skriftserie A nr 9. 1994.
7. Børn lærer af naturen. Information. 2001 13.06.21.
8. Vigsø BN, V;. Børn og Udeliv. Esbjerg, DK: CVU Vest Press; 2006.
9. Fjørtoft I. The Natural Environment as a Playground for Children: The Impact of Outdoor Paly Activities in Pre-Primary School Children. Early Childhood Education Journal. 2001;29(2).
10. Fyfe-Johnson AL, Hazlehurst MF, Perrins SP, Bratman GN, Thomas R, Garrett KA, et al. Nature and Children's Health: A Systematic Review. Pediatrics. 2021;148(4).
11. Lubans DR, Morgan PJ, Cliff DP, Barnett LM, Okely AD. Fundamental movement skills in children and adolescents: review of associated health benefits. Sports Med. 2010;40(12):1019-35.
12. Ammitzbøll; TPPSWPJ. Motorisk udvikling ved indskolingsalderen. Temarapport for skoleåret 2018/19. København: Databasen Børns Sundhed og Statens Institut for Folkesundhed; 2020.
